# Open Drug Discovery Teams: A Chemistry Mobile App for Collaboration

**DOI:** 10.1002/minf.201200034

**Published:** 2012-08-06

**Authors:** Sean Ekins, Alex M Clark, Antony J Williams

**Affiliations:** a Collaborations in Chemistry, 5616 Hilltop Needmore Road, Fuquay Varina, NC 27526, USA; b Molecular Materials Informatics, 1900 St. Jacques #302, Montreal, Quebec, Canada H3J 2S1; c Royal Society of Chemistry, 904 Tamaras Circle, Wake Forest, NC-27587, USA

**Keywords:** Apps, Chemistry, Cloud computing, Collaboration, Green chemistry, Mobile chemistry, Open data, Open science, Open source drug discovery, Web services

## Abstract

The Open Drug Discovery Teams (ODDT) project provides a mobile app primarily intended as a research topic aggregator of predominantly open science data collected from various sources on the internet. It exists to facilitate interdisciplinary teamwork and to relieve the user from data overload, delivering access to information that is highly relevant and focused on their topic areas of interest. Research topics include areas of chemistry and adjacent molecule-oriented biomedical sciences, with an emphasis on those which are most amenable to open research at present. These include rare and neglected diseases, and precompetitive and public-good initiatives such as green chemistry. The ODDT project uses a free mobile app as user entry point. The app has a magazine-like interface, and server-side infrastructure for hosting chemistry-related data as well as value added services. The project is open to participation from anyone and provides the ability for users to make annotations and assertions, thereby contributing to the collective value of the data to the engaged community. Much of the content is derived from public sources, but the platform is also amenable to commercial data input. The technology could also be readily used in-house by organizations as a research aggregator that could integrate internal and external science and discussion. The infrastructure for the app is currently based upon the Twitter API as a useful proof of concept for a real time source of publicly generated content. This could be extended further by accessing other APIs providing news and data feeds of relevance to a particular area of interest. As the project evolves, social networking features will be developed for organizing participants into teams, with various forms of communication and content management possible.

## 1 Introduction

Tools for drug discovery collaboration predominantly revolve around desktop computer applications^1^,^2^ though these authors believe that we will see a natural evolution toward the use of mobile apps in the drug discovery laboratory.^3^ Recently tools for selective sharing of chemistry and biology data have used “software as a service” (SaaS) as a business model e.g. CDD and HEOS.^1^,^2^,^4^ These software platforms have seen their user bases grow, especially in the neglected disease communities for tuberculosis and malaria. They can also be useful for the secure sharing of data with collaborators in which retention of intellectual property (IP) is important. We are increasingly seeing a shift to more companies, institutes and researchers openly sharing data. This again has been predominantly in the neglected disease space (e.g. GSK, Novartis and St. Jude′s sharing of malaria data).^5^–^9^ Alongside this there are increasing efforts by researchers to publish in open access journals and release data into open or free databases.^10^–^12^ In addition online tools are also being used to store science related content. Examples include FigShare,^13^ SlideShare,^14^ Dropbox^15^ etc. We should also not forget the efforts of various consortia to coordinate and make “big data” accessible. These include projects such as Elixir^16^,^17^ and Open PHACTS^18^,^19^ and a myriad of other initiatives to free up data that was once the sole preserve of the pharmaceutical industry.^20^–^22^ How can we possibly connect all of this data and impact research?

Social media platforms themselves can be useful for sharing data as evidenced by the success of blogs and wikis, both of which are commonly used as platforms for Open Notebook Science (ONS). These tools allow scientists to describe their scientific methods and results in near real time, link to other content such as uploaded images, graphs and models, among others^10^, ^23^ and do it all under open licenses.^24^ But how do you find these pioneering scientists that offer potentially valuable content? We believe the new efforts to create scientific mobile apps may have a role to play in collaboration.^3^,^25^,^26^

Another parallel concern is that of information overload in some subject areas: how are we expected to keep abreast of the avalanche of data flooding in from all sources? If you are a non-scientist, how do you find information on a scientific topic (besides using online search engines and referring to Wikipedia), connect with researchers, or even hope to make a contribution in a scientific area if you are a citizen scientist, biohacker^27^ or patient advocate? The following describes how three separate sources of inspiration have led us to create a new mobile app called Open Drug Discovery Teams (ODDT) for the sharing of open scientific data and, more specifically, bringing these tools to bear on the topic of drug discovery.

After one of us (SE) spoke with the parent of child with a rare disease, the parent shared that their experience post diagnosis suggested that they had to quickly become experts on a rare disease as quickly as possible. This involved finding and reading scientific papers on the topic (many of which were available via subscription only), and connecting with researchers around the globe to find out more about the disease. Ultimately, like many parents in a similar situation, this led to the foundation of their own non-profit to fund researchers, once they had found the scientists and clinicians that could perform the scientific studies necessary to help in their quest to begin to find a cure for their child. Additionally, we are seeing such parents also form companies as an additional way to expedite translation of the science for their rare disease to the clinic. This represents an example of what we are seeing on a large scale as there are over 7000 rare diseases. At the other extreme are neglected diseases which kill millions of people every year (e.g. tuberculosis and malaria), and although there is funding for research from foundations and governments, progress is slow and not well coordinated, with groups re-screening large libraries of compounds in whole cell systems (that have unbeknownst to them already been screened elsewhere) and only rarely using knowledge or computational tools that has been generated before or elsewhere. For these neglected diseases we are seeing a few open efforts to do drug discovery collaboratively for malaria and tuberculosis.^28^ Can we foster the same spirit in the area of very rare diseases?

A second source of inspiration was the ScienceOnline 2012 conference held in Jan 2012^29^ in which there were sessions on data overload and ONS. Various scientists described their strategies for dealing with the day-to-day challenge of overwhelming email, tweets, and other social media and news alerts etc. and trying to manage them all. The ONS discussion illustrated that many scientists were using their blog as an open notebook and then tweeting links from it to their contacts.

The third source of inspiration was the Pistoia Alliance.^30^ This is a pre-competitive initiative in which pharmaceutical companies and software vendors are attempting to develop standards that can be used by all companies for their software systems and, where appropriate, developing software tools collaboratively rather than each pharmaceutical company developing its own informatics tools.^31^ In February 2012 they held a reception at the Royal Society of Chemistry with attendees from pharmaceutical companies, software companies, and venture capital funders. As an ice breaker they organized a Dragon’s Den^32^ type competition in which they wanted to gather ideas of a new external “R&D Service” that would transform the Pharmaceutical R&D sector and that would be in production and fit-for-use in 2015.

The intersection of all of these sources of inspiration encouraged us to participate in the Dragon’s Den. The following describes the ideation, software development, feedback and ideas for future development of ODDT. In keeping with the openness espoused in this article we shared our concepts and ideas in blogs including the incremental development and work in progress iterative reports associated with the technology development.^33^–^35^

## 2 Methods

### 2.1 Ideation

**Figure 1:**
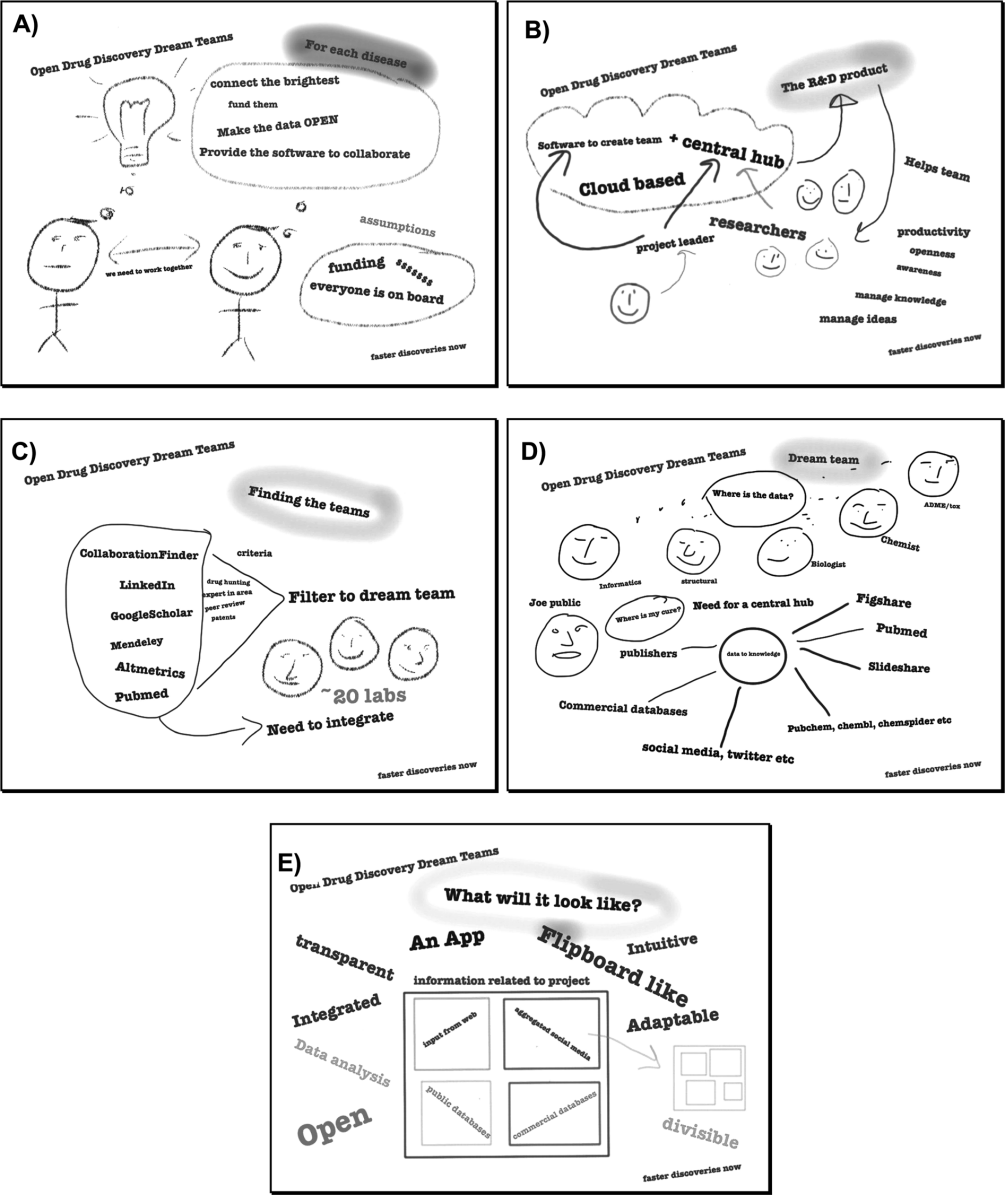
How the initial idea for ODDT was sketched out around core concepts. A) Connecting scientists. B) Cloud-based software to help collaborations. C) Integrating tools to find the right scientists. D) Integrating sources of social media, open and commercial data in a central hub. E) How the mobile interface could integrate the data and be perused.

Using an iPad with the Sketchbook Mobile Express app (Autodesk, Inc.^36^) one of us (SE) sketched out several slides of ideas (Figure 1 A–E) which laid out the concept of one finding the best collaborators to do open research and then developing a tool to connect the open data and the researchers. Although these slides are perhaps simplistic they were a modern day “back of an envelope” sketch that connected the basic concepts from all the sources of inspiration. After sharing these slides with the co-authors it was decided to develop a prototype for the Dragon’s Den competition that, initially, would harvest the Twitter feeds on a fixed number of specific research topics.

### 2.2 Development

One of us (AC) has developed a mobile chemistry aware platform (the *Mobile Molecular DataSheet*) which implements powerful cheminformatics features. The core functionality is based on solving the problem of providing a high quality chemical structure editor for a palm-sized mobile device with highly constrained input functionality.^37^ This product has since matured and added extensive derived functionality, such as reaction editing, datasheet management, web-services access and collaboration tools. The functionality has been packaged in library form, and used as the basis for a number of other apps with more task-specific functionality.^38^

### 2.3 Client App

The primary user interface is via the ODDT app, for iOS-based devices (iPhone, iPod and iPad). The app provides a user interface that is inspired by the “magazine-like” Flipboard app:^39^ the user initially selects from a list of topics, and from there can flip through recently posted content. The app is free for anyone to use, and provides content-consumption features as its primary purpose. As it evolves, the app will also be used to participate in active content-sharing and social networking activities.

### 2.4 Server

The server-side architecture is based on the *com.mmi* software stack, used by the *molsync.com* service, which currently provides additional cheminformatics capabilities for various mobile apps.^40^ The extensions designed to host the ODDT project make use of Twitter as the primary source of content, which is regularly polled and assimilated into the data collection. The service provides an API for accessing ODDT topics and content. As the project evolves, the server will be gradually augmented to recognize particular data sources and information streams, and provide value added functionality. Currently it is able to recognize chemical data such as molecular structures, reactions and datasheets.

### 2.5 Development Milestones to Date

The ODDT project is designed so that it can be ramped up on a gradual curve and has provided useful functionality from the beginning. Because the content source is initially derived from Twitter, which consists entirely of public data, has a public API, and has a huge existing user base, the ODDT project can demonstrate initial utility by providing added value based on the existing data stream.

We initially defined a small number of topics, corresponding to Twitter hashtags such as: #tuberculosis, #malaria, #hivaids, #huntingtons, #sanfilipposyndrome, #leishmaniasis, #chagas and #greenchemistry. We have built the server architecture for harvesting URLs from these tweets, and gathered them together into a collection that can be accessed via an API. We have performed some minimal recognition of the links and, in particular, annotated those which lead to chemical data content, as well as enumerating a list of embedded images within the corresponding web page. We have also hosted the server on molsync.com initially, with the intention of relocating at a later date.

The client app, with its “Flipboard-like” interface, is at a minimum viable product stage: it provides browsing functionality for the topics defined by the ODDT project. The content for each topic roughly corresponds to individual tweets with hashtags matching the predefined subject areas. Links that lead to chemical data (e.g. molecules, reactions, datasheets) are viewable using cheminformatics functionality embedded into the app, while actual editing is possible by opening the content using other apps installed on the device.

Other links are currently treated as generic web pages: linked images are shown as thumbnails, and each page can be viewed with an inline web browser. This represents a first in class prototype mobile app for aggregating open data on scientific topics and diseases that are rare or neglected in particular. The app is currently able to make use of the operator′s Twitter account(s) to emit endorsements and comments for content items, which are subsequently harvested by the server in order to rank content and decide which documents should be retained in the system. This serves as a basic crowdsourced curation method, which will be expanded upon. During this prototype version the method for users to add content to ODDT is to use a standard Twitter client to emit links to relevant content, and use one of the topic hashtags. The client app and server architecture do not, at this stage, identify team members or provide any direct ways to influence content, other than rating or commenting on it. An app from *Molecular Materials Informatics*, *MolSync*, has been enhanced to make it particularly straightforward for users to inject chemical data into any of the topic streams.

### 2.6 Testing and Feedback

We initiated an alpha version of the software providing it to a dozen or so researchers with an interest in testing it. This provided an opportunity to address usability issues, fix bugs, and observe use patterns for scientists with a diverse range of experience with mobile and social networking software. Following the conclusion of the alpha test, we submitted the beta version to the iTunes AppStore, with the intention of growing the number of test users by making it freely available to anyone with an iOS-based device.

### 2.7 Support and Training

The free app will not have any official technical support, though unofficially we are very interested in the users′ experience with the app, both positive and negative. Responses from users regarding issues, bugs, and popular features will strongly influence the evolution of the product. As the community grows, the inherent communication features of the app will provide an intrinsic support group. The free app will ultimately have videos and documentation online at the project website, *oddt.net*, and at *scimobileapps.com.*^41^,^42^

## 3 Results

**Figure 2:**
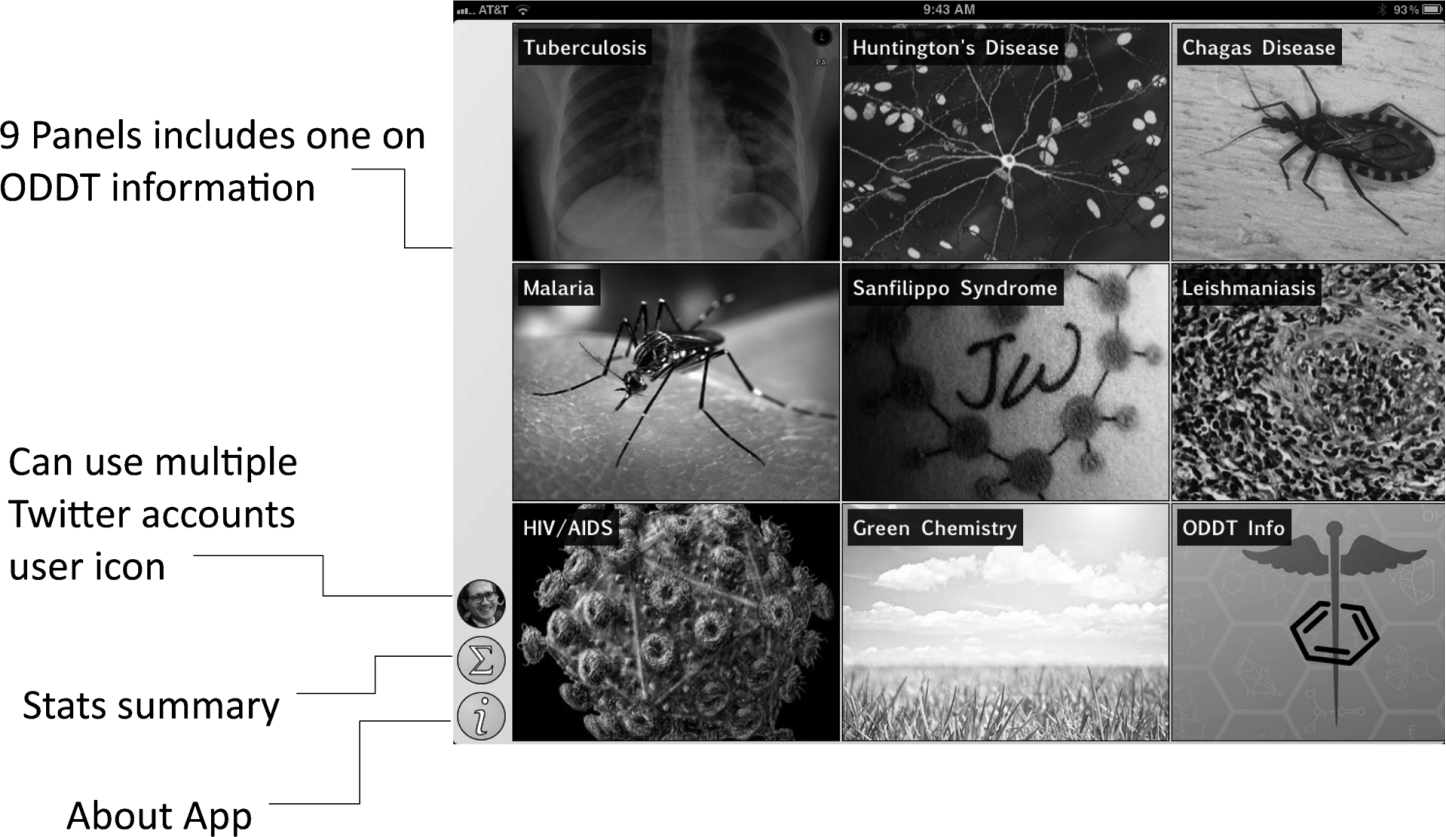
Entry screen for ODDT. 9 topics are shown, which can be tapped to open content. The side bar has other informational icons.

**Figure 3:**
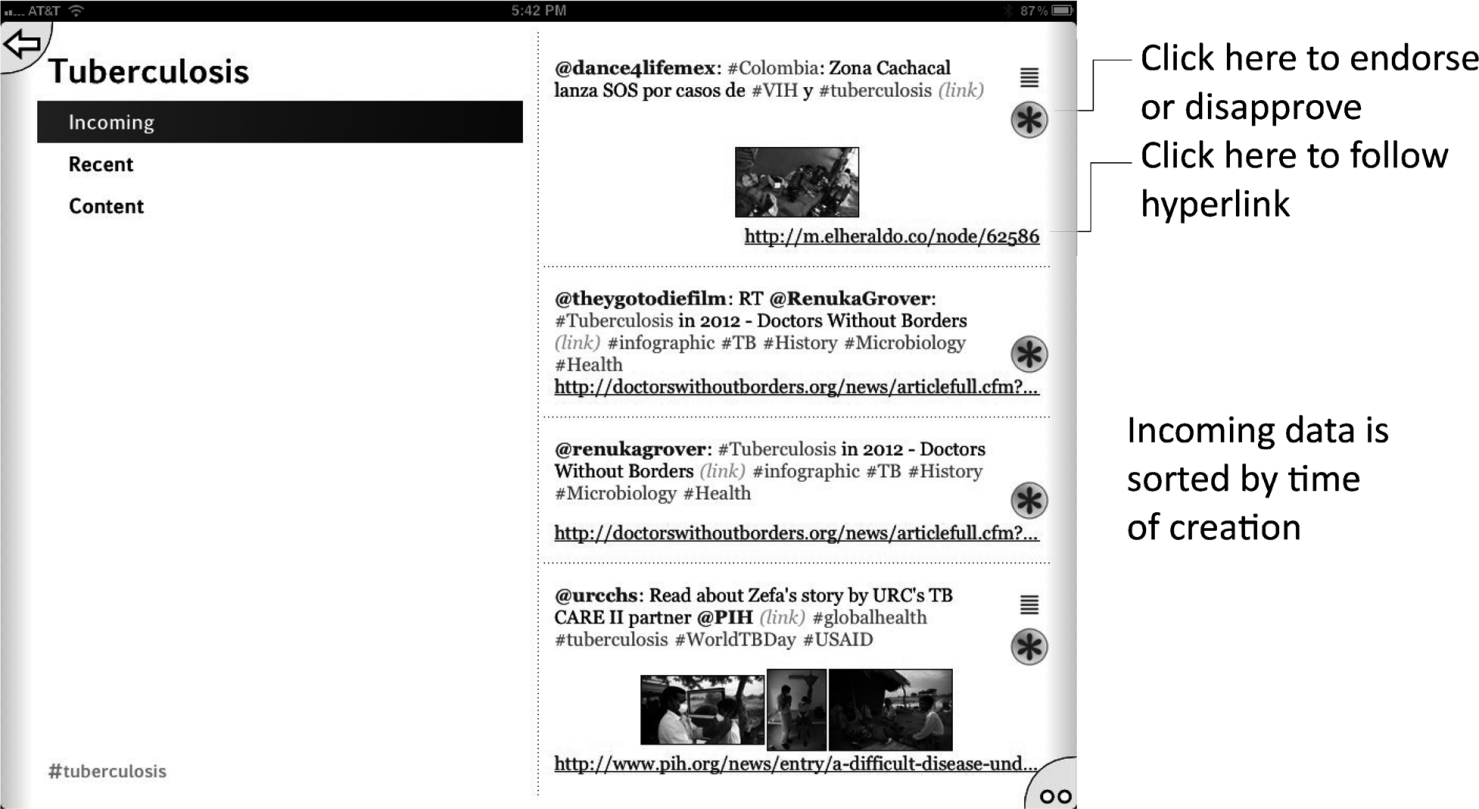
Tuberculosis topic page listing incoming content. Factoids are shown with hyperlinks and a voting button.

**Figure 4:**
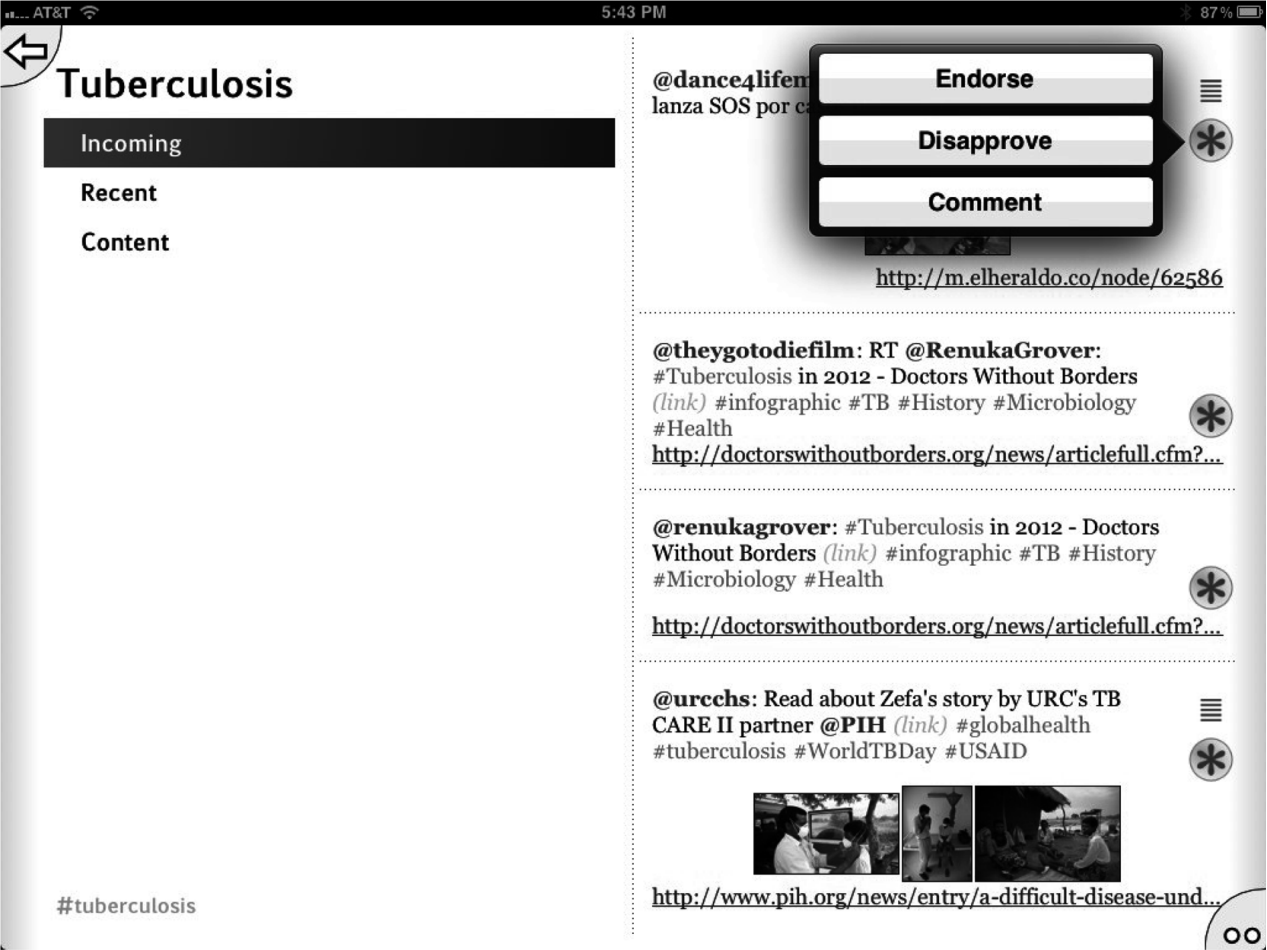
Tuberculosis topic page listing incoming content showing voting options.

**Figure 5:**
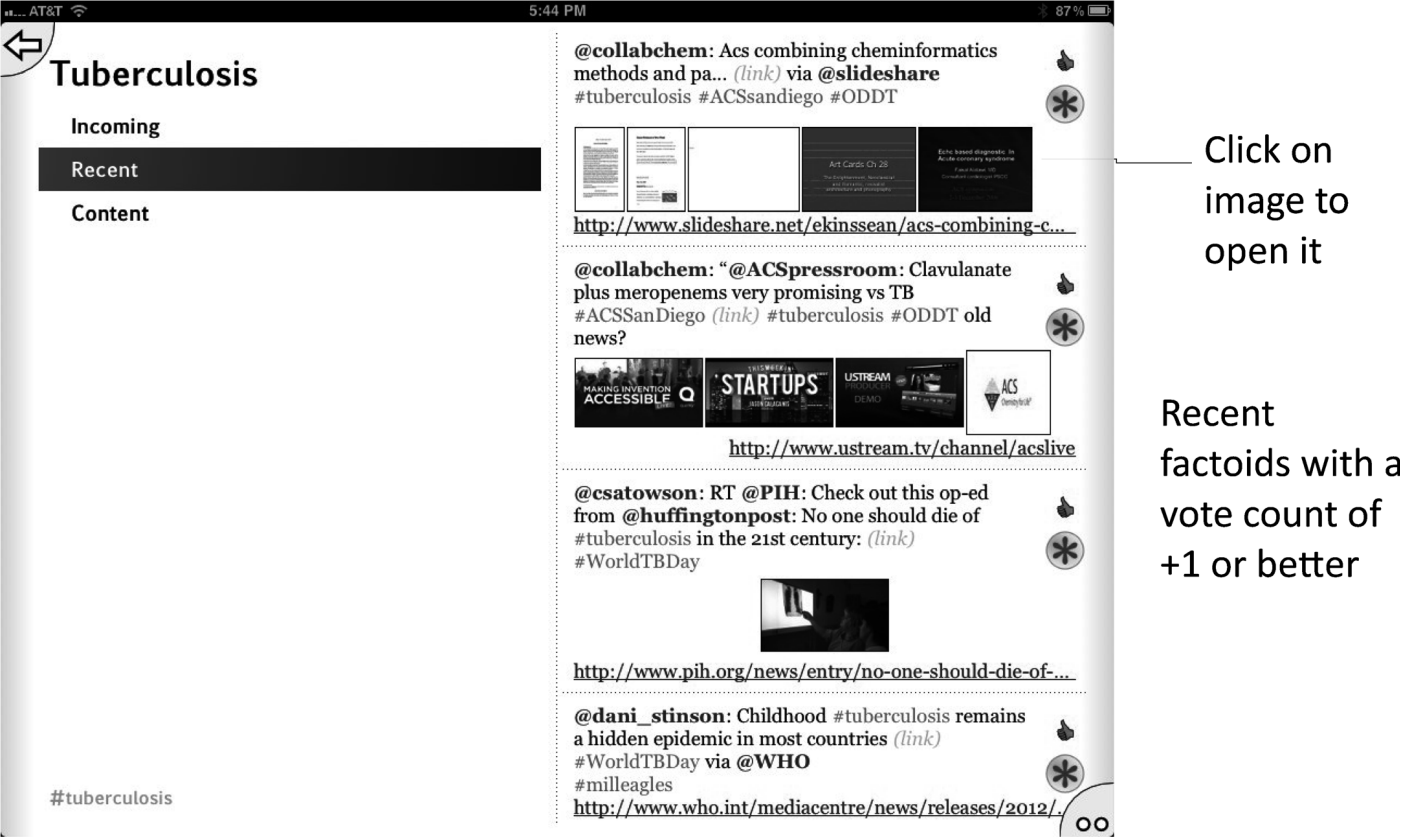
Tuberculosis topic page showing recent factoids with one or more vote.

**Figure 6:**
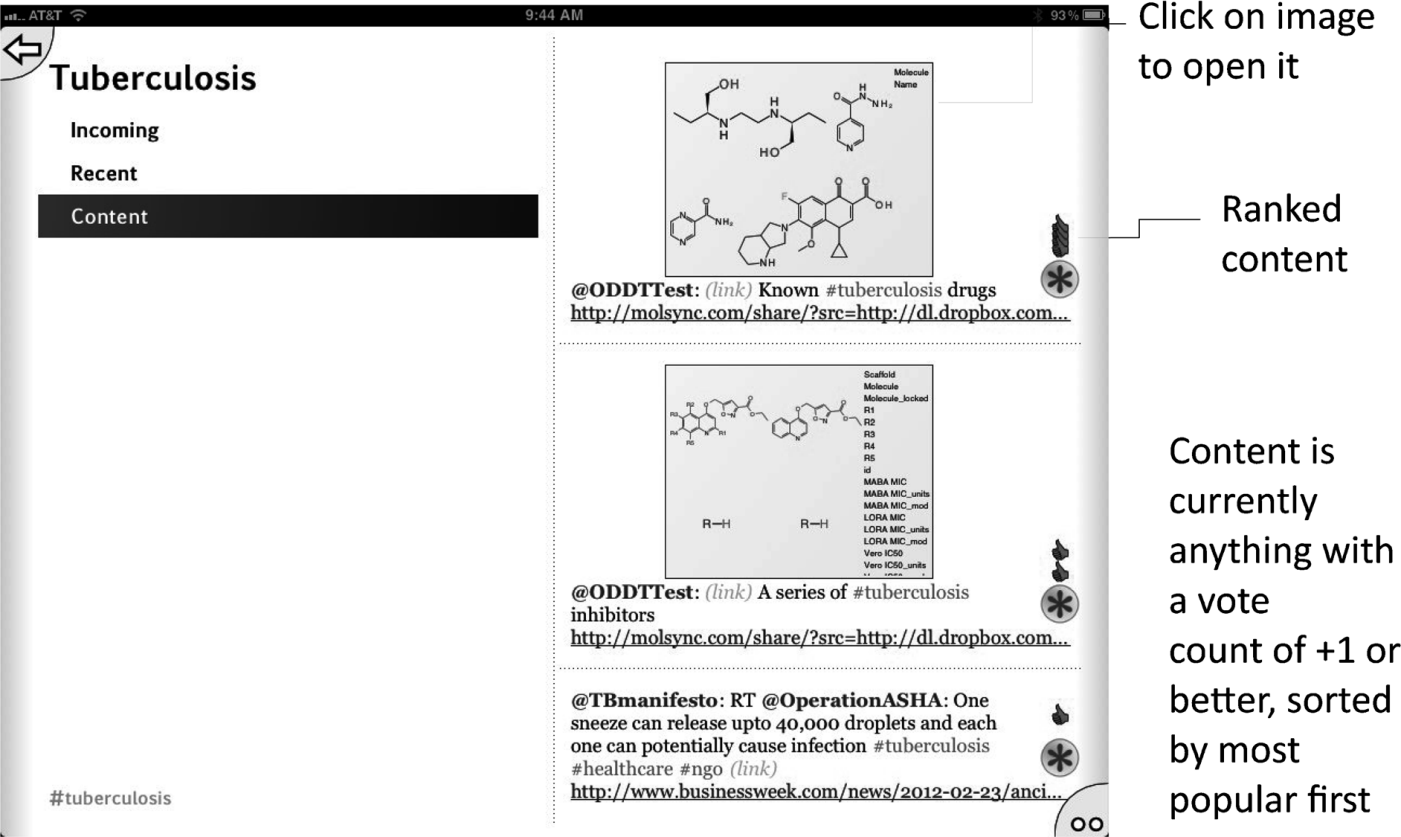
Tuberculosis topic page showing content factoids sorted in order of most votes in favor.

**Figure 7:**
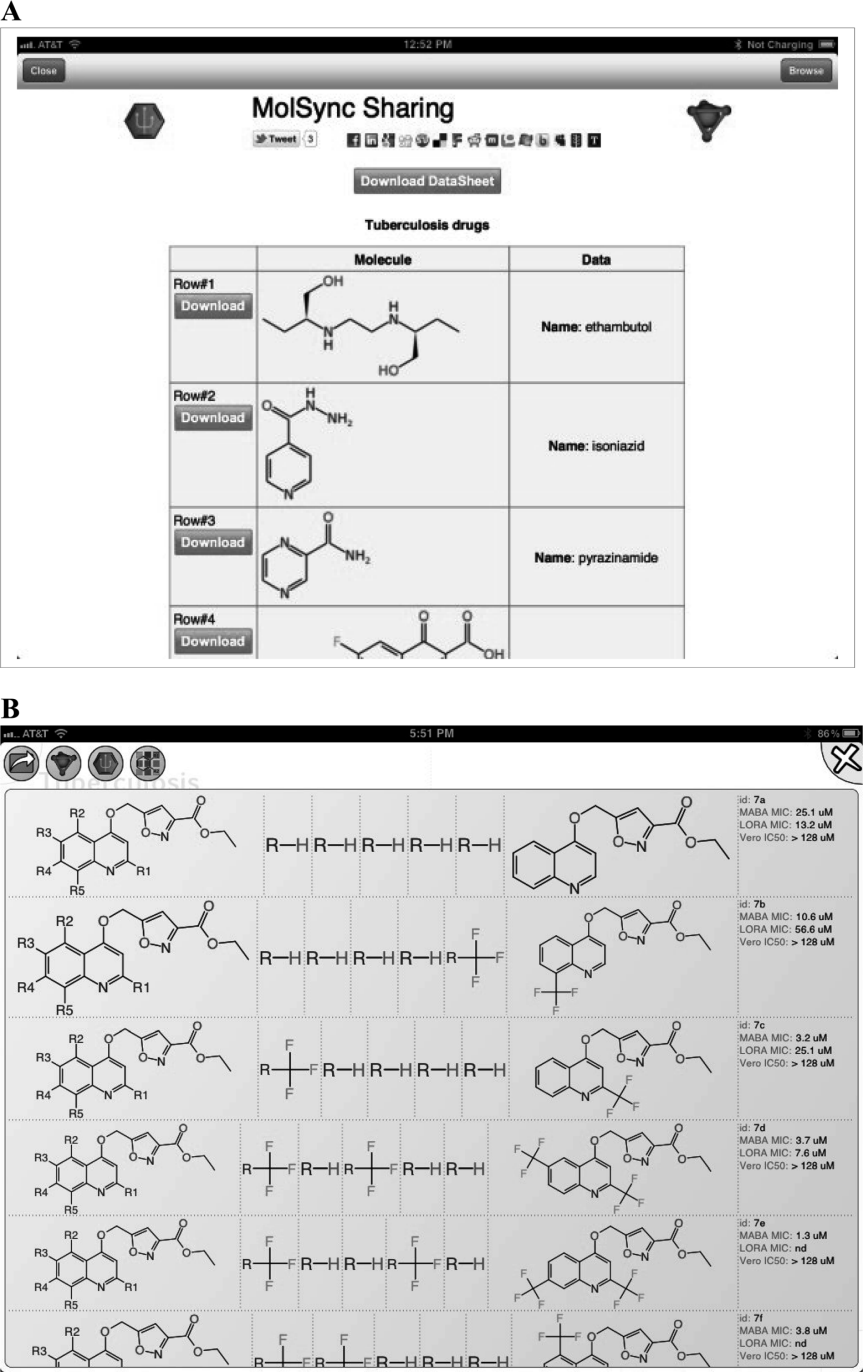
Examples of links out from the ODDT app. A) A set of tuberculosis drug structures from the MolSync app,^70^ B) A structure activity table from the SAR Table app.^71^

**Figure 8:**
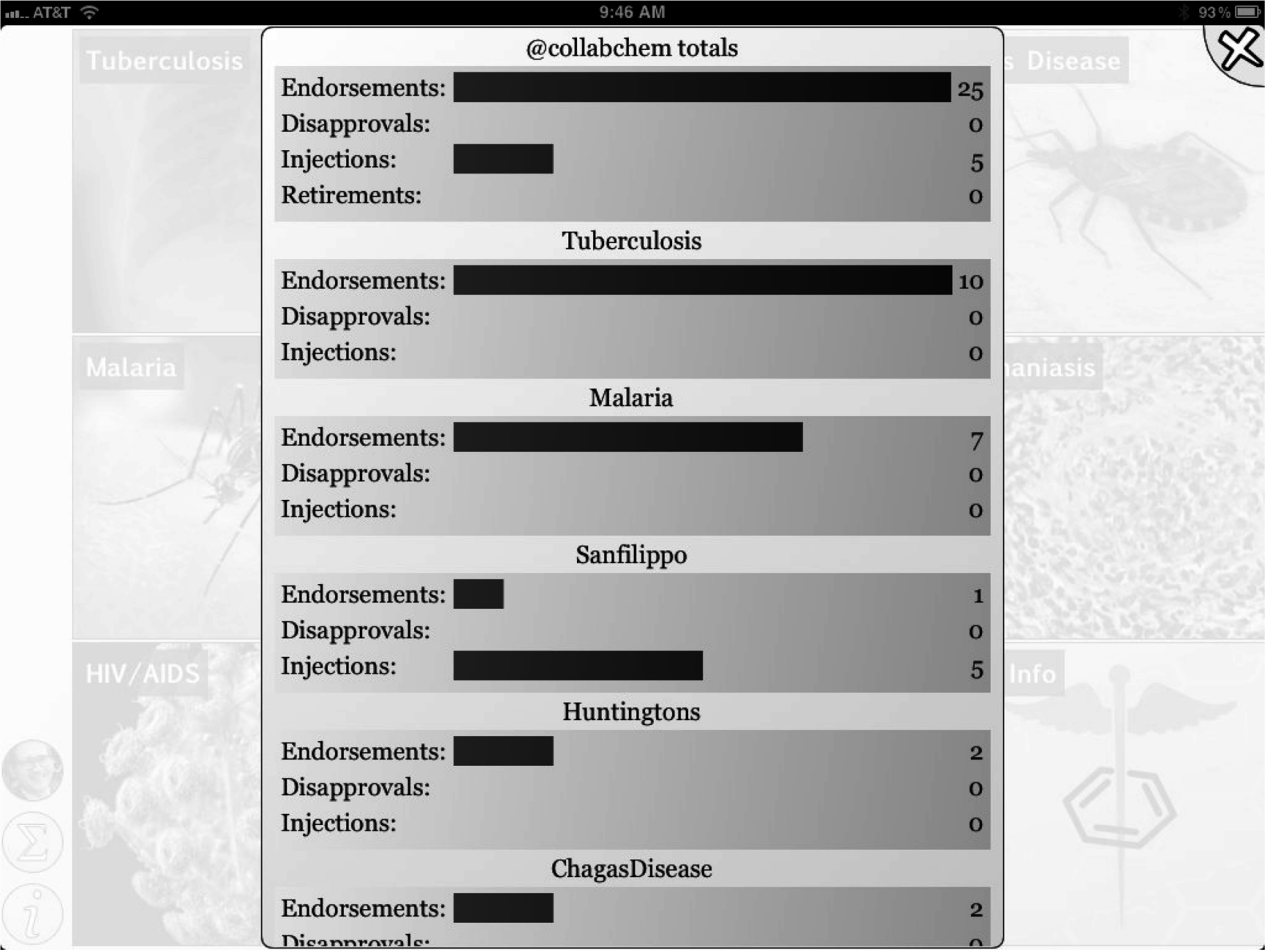
Statistics summary, showing endorsements, disapprovals, injections and retirements.

Initially we used the app to harvest Twitter feeds on the hashtags described above for the diseases: malaria, tuberculosis, Huntington’s disease, HIV/AIDS, and Sanfilippo syndrome as well as the research topic green chemistry,^43^ which is of interest because its community is highly receptive to open collaboration. We have since added #H5N1 (bird flu) and #hh4gan (Giant Axonal Neuropathy). All of these subjects have high potential for positively impacting the research environment using computational approaches and dissemination information via mobile apps.^44^,^45^ We have used Twitter to feed content into these topics, by providing links to molecules and links to structure-activity tables. This minimal app was described at the Pistoia Alliance Dragon’s Den meeting along with a few of the initial concept sketches. Since then we have added the ability to endorse or reject tweets, expanded the number of topics by adding Chagas Disease and Leishmaniasis as well as a topic for information on ODDT. In addition the ability to visualize a thumbnail image for each tweet was added, as well as recognition of linked images. The app can now be used to manage multiple twitter accounts for the user. We also added the ability to summarize the endorsement and rejection activity for the user, arranging the topics on the screen from most to least used (Figures 2–8). The entry screen to the app displays the topics ranked by use (Figure 2). Tapping a topic image opens the topic browser, starting with the *incoming* page. Newly added content is listed on the right (Figure 3). Each tweet that has been introduced into the data collection is referred to a *factoid*. A factoid can be endorsed or disapproved (Figure 4), and the hyperlinks can be followed. The *recent* page shows factoids with one or more vote (Figure 5) while the *content* section shows the most popular voted content in rank order (Figure 6). Molecule thumbnails can be tapped to open in other apps like *MolSync* (Figure 7A) or *SAR Table* (Fig. 7B). Back on the entry screen, tapping the statistics summary button opens a listing of endorsements, disapprovals, injections and retirements for each hashtag (Figure 8). We have described this work at several meetings including the American Chemical Society^46^ and will continue to promote its use in the scientific communities appropriate for each hashtag. From app idea to submission to the iTunes AppStore took a little over 2 months.

## 4 Discussion

We are witnessing the growing menace of both increasing cases of drug-sensitive and drug-resistant *Mycobacterium tuberculosis* strains and the challenge to produce the first new tuberculosis (TB) drug in well over 40 years. Tuberculosis kills 1.8 million people every year (∼1 every 8 seconds).^47^ This is equivalent to malaria in terms of morbidity and it is believed that one third of the world’s population is infected. Drug-drug interactions and co-morbidity with HIV are also important for TB. Recent years have seen increased investment from the Bill and Melinda Gates Foundation (BMGF) and the National Institutes of Health (NIH) but this has not (as yet) had a big impact on new clinical candidates and of those identified the majority of these are coming from pharmaceutical companies.^48^ The clinical TB pipeline is therefore thin and weak.^49^ Compounding this is the lack of data sharing on the scale of malaria where significant efforts have been made to assure the availability and access to data.^5^–^8^ There are already open source efforts for drug discovery for TB^50^–^52^ and malaria^28^ in India (OSDD) but this has no real international traction yet. In the USA there are disconnected efforts for collaborative TB drug discovery, including those of Collaborative Drug Discovery,^1^,^2^,^53^,^54^ pharmaceutical companies, academia and NIH. BMGF does not extensively fund European efforts while FP7 grants fund the More Medicines for Tuberculosis (MM4TB) project^55^ as a collaborative pharma-academia collaboration (but this is a closed initiative between those groups involved). The result is that there is significant TB data hoarding and scientists are not learning from the failures of others resulting from these already well-funded efforts. We believe that there needs to be a change by providing the neglected disease community with a way to make more data and content available on these diseases. This includes access to scientific papers or the key results and molecules.

Traditionally, rare (orphan) diseases have very small patient populations compared with neglected diseases, though there is no global agreement on what this size is. In the US it is described as a disease that affects less than 200 000 people. Some estimates suggest this category includes over 7000 rare diseases affecting 25–30 million people.^56^, ^57^ Even though over 300 orphan drugs have been approved since the passage of the Orphan Drugs Act in 1983, there is still a long way to go until most rare diseases have a treatment.^56^,^57^ The recent publicity around the ULTRA act and TREAT act legislation suggests there is considerable interest to promote more research into rare diseases and translational research^58^,^59^ and that this may lead to commercial opportunities. We believe the field of rare diseases needs increased collaboration and open sharing of data in order to facilitate discovery of new therapeutic approaches.

At the same time we are seeing that drug discovery is shifting focus from the industry to outside partners and in the process creating new bottlenecks, suggesting the need for a more disruptive overhaul. With new mobile computer hardware and software applications, aspects of drug discovery will be done anywhere by potentially anyone, while emerging technologies could be used to reimagine the drug discovery process.^60^

Whether one mobile app like ODDT can be used to revolutionize the way we perform neglected and rare disease research, and in turn the pharmaceutical industry, remains to be seen and we perhaps are quite early on in the process. We have however successfully put together a novel tool for open drug discovery collaboration that did not exist previously and leveraged social media.

Stimulated by the Pistoia Alliance Dragon’s Den experience it is important to have a clear value proposition which can be summarized as follows. *The project is intended to bring together freely accessible and open data in a single aggregated collection, and then facilitate the forming of open research teams around this data. Such teams consist of individual researchers in academia and industry, as well as other interested parties, including the general public, who have a desire to be kept up to date with research in a particular subject (disease) area*.

By assembling a focused group of disparate individuals and organizations scattered about the globe, the opportunity exists to disseminate important information to a highly relevant target audience. This includes information about new scientific developments of interest, to network and discover other researchers with complementary interests, and opportunities to collaborate in other ways such as highlighting conferences, publications or laboratory data sharing.

As the software collection evolves and improves in its capabilities, data from open science will be integrated and made usable within a common framework. Team members will be able to borrow and reuse a growing collection of existing data. Networking and collaboration features will be introduced, allowing researchers to post incomplete or speculative data, while other researchers fill in missing details, and the community as a whole can debate, contest or endorse data based on its quality. ODDT may also contribute to the nascent development of “nanopublications”.^61^ The app could also be used as a type of open lab notebook whereby individual researchers share links (URLs) to content and the app aggregates these.

**Figure 9:**
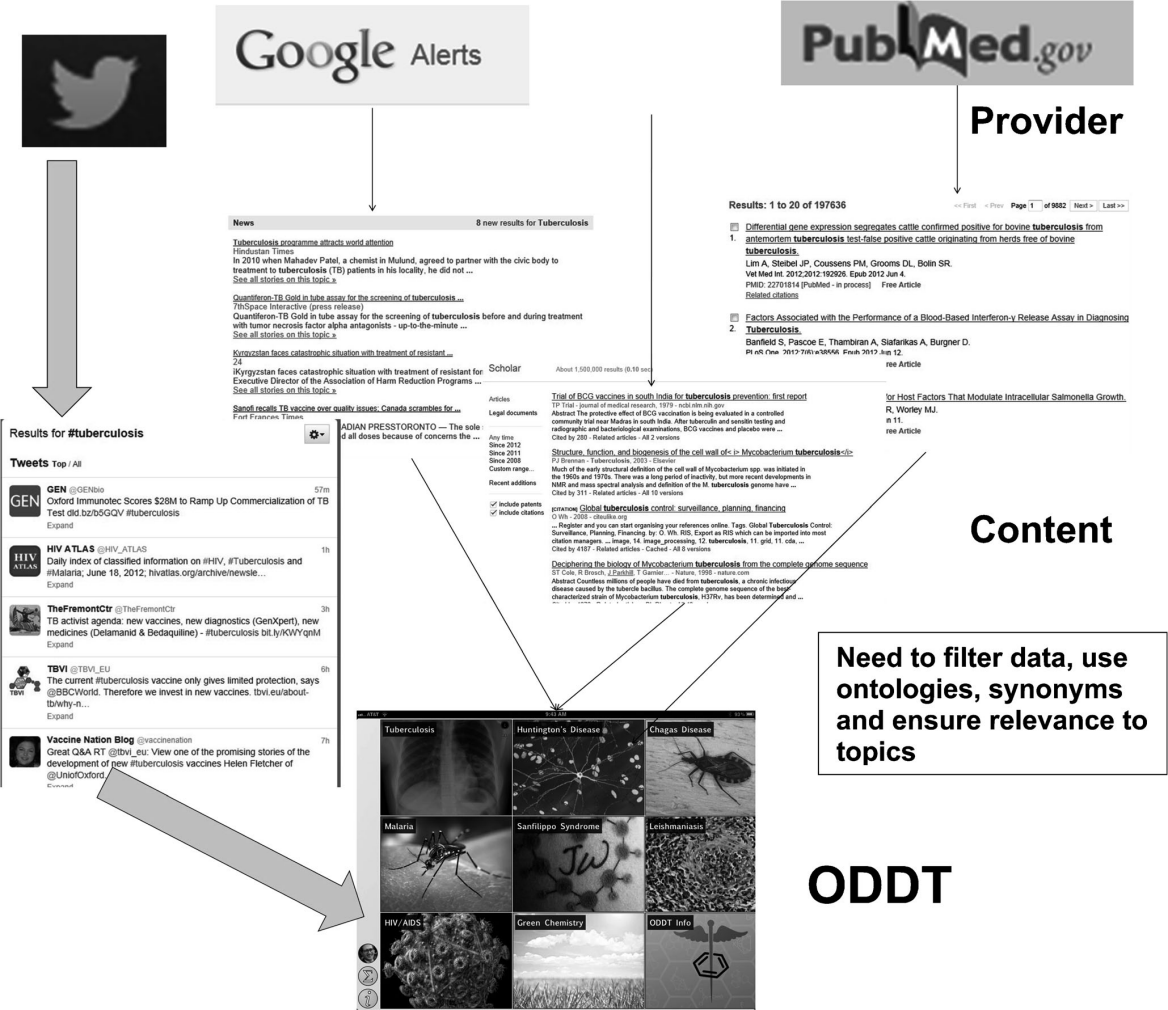
Integrating additional data sources in future (thin arrows).

We certainly envisage that we could capture data from other sources, and are actively considering population of the ODDT data collection with information from sources such as Google Alerts,^62^ Google Scholar,^63^ PubMed,^64^ and any other service that provides an API and a preliminary method for categorizing scholarly works (Figure 9). As ODDT expands we will require ontologies and data curation (automated and manual) as well as other steps to filter data and ensure relevancy for the topic. We are also considering means by which new topics can be created by user consensus, though in the early stages of the project while the user community remains small, new topics will continue to be added manually. Recently we have started a crowdfunding campaign using IndieGoGo^65^ to assist in the integration of additional data sources and the donor rewards are the selection of additional rare diseases.

We may in future introduce a number of additional types of annotations that can be added to a factoid. Assuming that the factoid originated from a link to a core document, these annotations can be used to define commonly expected subsections, in a somewhat structured format. Some of the annotation types we are considering are:

– **Description**: A title-like summary of the content.
– **Synopsis**: An abstract-like paragraph summary of the content.
– **Comment**: A short comment discussing the content′s relevance or lack thereof.
– **Review**: A detailed analysis of the content, meant to evaluate it critically and inform readers of what can be learned from it, and any caveats that the reader should be wary of.
– **Subtopic**: A classification within the topic, to encourage grouping.
– A keyword for subsequent classification and recall.
– **Figure or image**: A graphic, possibly with a caption. Typically provided as a web standard image format, e.g. SVG, PNG.
– **Table**: A data table of some form, loosely defined. A wide range of formats, often machine interpretable, e.g. delimited text, Excel, etc.
– **Chemical data**: A highly structured data bundle that represents some collection of chemical structures, reactions, auxiliary data, and other well defined content that is machine interpretable.

These annotations can come from a variety of different users, and may be duplicates. When compiling optimally presentable results, it may be necessary to curate these by way of a priority system.

While this system is nominally intended for annotation of content that is fully accessible via the original URL, it is potentially quite useful to annotate documents to which many users do not have access, e.g. articles that are behind a journal pay wall. The annotation of journal articles is already possible using exemplar document readers such as Utopia documents.^66^ The collective annotations of the users who have access to the article may provide enough information to allow some users to bypass the purchasing of the article. It also has potential as a path to assemble articles from scratch, in a fully open medium, and institute a kind of informal peer review. In addition this functionality goes further towards an open notebook use case scenario in which the scientist publishes their results whenever a suitable point is reached, whether or not the results are sufficiently complete to warrant dissemination in a traditional peer reviewed article.

Users of ODDT can either be passive (lurkers, browse only) or active (crowdsourced addition and curation of content). The cumulative curation efforts of active users are what will make the project successful, and so these activities should be encouraged and rewarded as much as possible. Because all of the information flows through Twitter, which is public, the users gain peer recognition via their Twitter accounts but it is also possible that the participation could be measured using Alt-Metrics^67^ approaches like Total Impact^68^ and Altmetric.^69^ ODDT also tracks statistics on user activity associated with each Twitter account. By designing a set of activity milestones and assigning badges or trophies for activity overall, or a ranking system for individual topics, a kind of “compulsion loop” can be designed to keep users interested in continued participation.

Curated content from ODDT can be compiled from its raw sources, and combined with the various kinds of crowdsourced input, and periodically produced as an online “booklet or review”. To a large extent it will be possible to do this in an automated fashion, but some manual oversight and editing may be required. If sufficient production levels are able to be maintained, access to these “booklets” could be made available on a fee-based subscription basis. This fee ought to be waived for users who participated in curation at a specified badge/ trophy level over the period.

Since the ODDT data source consists of incoming links to arbitrary data, some recognition of particular data types is valuable. From early versions, links from *molsync.com*^70^ and *chemspider.com*^11^ have been recognized, and their chemical data extracted even though the links refer to standard HTML pages. A number of other open resources are available on the internet, and extracting the data in machine readable form, as well as standard renderable web pages, increases the value of the ODDT project. New recognition types will be added regularly, and some explicit collaborations are to be anticipated and we welcome additional suggestions.

The server for the ODDT project initially only stored minimal information – not much more than the relevant tweets that make up the data collection. The payload content is typically referred to by way of a link, which is passed through to the client and downloaded as necessary. In a later version, chemical data (structures, reactions, property and activity data) will be parsed by the server, and stored within the ODDT database. This growing chemical data warehouse will provide fast access to associated chemical content, and searching capabilities. There are a vast number of value added features that can be built on top of such data. The rate limiting step is ensuring that the database has sufficient capacity, and has sufficiently high performance to handle a growing data warehouse, which can sustain complex queries. This may require the addition of extra servers, which would be expedited by securing a source of funding for the project. This is another of our aims with the crowdfunding through IndieGoGo.

Later versions of the software could integrate with other cheminformatics and drug discovery related apps to be developed (e.g. structure searching, activity data extraction, structure-activity series creation, automated model building, docking against known targets, pharmacophore hypothesis generation etc.). One could imagine that the app will be able to function as a virtual research organization in which it becomes the primary tool for sourcing data, sharing data, models and hypotheses as well as selective collaboration with other scientists in public through the open internet, or in private behind a company firewall. ODDT could then be considered a foundation for crowdsourced drug discovery.

With investment resources we intend to adapt the product for licensing to pharmaceutical research institutions for internal use and make it a closed app. Private documents can then be mixed with external “open” data to create a valuable internal resource. We will leverage the ongoing development of software for analysis of documents and chemical data to provide informatics capabilities for content discovery and extraction. These capabilities will complement then rapidly displace existing chemical database software as mobile and cloud computing becomes the accepted paradigm for drug discovery.

ODDT can already be used by scientists to make others aware of, for example, molecules that are described in a research paper that others may not be aware of because of accessibility issues with a paper. ODDT could also be used as a repository for preprints on a particular topic, thus making data and research more freely available. We have demonstrated the viability of the approach by tweeting in content ourselves and adding the #ODDT hashtag to auto-endorse it. The project is open to participation from anyone, and much of the content is derived from public sources, but is amenable to commercial data input (from publishers and pharmaceutical companies). In future versions we imagine the user will need to be able to select their research topics of interest, corresponding hashtags and images for use in the app.

To date we have performed this work without funding and made the app freely available. We would be happy for foundations, societies, individuals and companies to fund or sponsor research topics either directly or through our crowdfunding campaigns.^65^ We hope that scientists will increasingly tweet links to papers, blogs, slides and molecules of relevance to scientists following the topic hashtags.

## Abbreviations

MMDS Mobile Molecular Data Sheet
ODDT Open drug discovery teams
SAR Structure Activity Relationship
URL Uniform Resource Locator.

## Conflicts of Interest

Sean Ekins consults for Collaborative Drug Discovery, Inc. and is on the Board of Directors of the Pistoia Alliance. Alex M. Clark is the owner of Mobile Molecular Informatics, Inc., which has produced the software libraries used by the ODDT app. Antony J. Williams is employed by The Royal Society of Chemistry which hosts the ChemSpider database discussed in this article.
